# Three-Dimensional Finite Element Study of Endodontically Treated Maxillary Central Incisors Restored Using Different Post and Crown Materials

**DOI:** 10.7759/cureus.33778

**Published:** 2023-01-14

**Authors:** Nour Al-Deen Kharboutly, Mirza Allaf, Shaza Kanout

**Affiliations:** 1 Department of Fixed Prosthodontics, Faculty of Dental Medicine, Damascus University, Damascus, SYR

**Keywords:** zirconia post, glass fiber post, cast metal post and core, stress distribution, post and core, 3d finite element method, computer-aided design and computer-aided manufacturing (cad/cam)

## Abstract

Background/purpose: Restoring endodontically treated teeth is a common problem in dental practice. Post and core restorations are one of the major options in the rehabilitation of these teeth. However, there is no final decision regarding the best material or technique to be used with these restorations. So, this study aimed to evaluate the effect of different post and crown materials on the biomechanical behavior of restored maxillary central incisor using the finite element method.

Materials and methods: A total of 10 3D models of endodontically treated maxillary central incisors restored with two prefabricated posts and three custom-made posts were modeled and grouped according to post material (gold, nickel-chrome, zirconia, and glass fiber) and crown material (lithium disilicate, and zirconia). Finite element analysis was conducted, and stress distribution was evaluated using von Mises criteria.

Results: Both crown materials showed stress concentration at the force application site mainly on the intaglio palatal surface of the crown. However, more stress values were observed within zirconia crowns. All posts showed stress concentration at their buccal sides. However, more stress values were observed in zirconia and metal cast posts compared to glass fiber posts that transfer more stress to root dentin.

Conclusions: Post and crown materials affect the stress distribution in the tooth-restoration complex. Using high elastic modulus posts slightly decreased stress in root dentin despite concentrating more stress within their structure. However, glass fiber posts resulted in more homogenous stress distribution in the tooth-restoration complex. Crown material did not influence the stress distribution in root dentin. Custom-made posts decreased stress within crowns, regardless of the crown material. However, more stress values were observed within zirconia crowns. Custom-made zirconia posts and cores showed a similar stress distribution as non-precious metal cast posts, so they may be used as a suitable option where esthetic is desirable.

## Introduction

Using posts and cores foundations is the most common procedure in restoring endodontically treated teeth. Many materials and techniques had been suggested for this purpose. However, there is no final decision regarding the best material and technique to be used with these restorations [1].

Endodontically treated teeth exhibit major loss of coronal dental tissues due to caries, fractures, and access cavity preparation procedures [2]. Clinicians usually use posts and cores foundations to support the final restorations. Root canal posts are mainly used to retain cores and restorative crowns [3,4]. Posts are usually classified into two major categories: prefabricated and custom-made [5].

Prefabricated posts are mainly classified according to post material into high elastic moduli metal posts like stainless steel and titanium posts, non-metal posts like zirconia, and low elastic moduli fiber-reinforced composite resin posts like carbon, glass, and quartz fiber posts [6]. Most prefabricated posts exhibit circular cross-sections which may contribute to the misfit and thick cement layer between the post and the root canal walls, especially in oval and flared canals, thus increasing the risk of post-debonding [7]. On the other hand, the possibility of saving time because of applying the post and core chairside and the elimination of lab procedures are the major advantages of using prefabricated posts [8].

Custom-made posts are traditionally fabricated in dental labs from cast metals like gold, palladium, silver, and base metal alloys [5]. However, recently they are fabricated using Computer-aided design/Computer-aided manufacturing (CAD/CAM) technology from materials like zirconia, glass-fiber reinforced composite, cobalt-chrome, hybrid ceramics, nano-ceramics and Polyetheretherketone (PEEK) [9].

Custom-made posts adapt well to the root canal, have good mechanical proprieties, and have a long history of success in restoring endodontically treated teeth [10]. They can withstand high occlusal forces before deformation occurs because of their high modulus of elasticity [11,12]. However, some studies reported that cast posts may cause root fractures because of their rigidity [13]. In addition, the prevalence of highly translucent ceramic crowns raises a concern regarding their esthetic proprieties. So, in response to this concern, nonmetal posts were developed such as glass fiber posts which became increasingly more popular because of their esthetic and elastic properties which may contribute to better stress distribution and decrease the risk of root fractures because of their proximity to dentin elastic properties [14].

Advancements in dental materials and CAD/CAM technology enable us to fabricate less time-consuming, highly accurate esthetic restorations [15]. Posts and cores were not an exception as CAD/CAM technology permits fabricating of highly esthetic custom-made zirconia posts and cores [16,17] which can be used with full ceramic crowns thus overcoming the esthetic limitations of cast posts and cores. Moreover, as a one-piece post and core, the post-core relation effectively supports the final crown and overcomes the problem of post-core debonding which may be happened with prefabricated posts [18].

The literature suggests many factors that could possibly affect stress distribution in endodontically treated teeth like the post and core material, crown material, cement, post length, post diameter, and remaining dentin [12,19]. Some authors studied the relationship between the cement in the post-dentin interface and the post-core restoration success. They obtained the best results with 0.2-0.3 mm thicknesses [19]. Whereas other authors reported 0.1-0.3 mm as the optimum thicknesses [20]. Cement thickness is hard to control clinically and its effect on stress distribution is minimal [19].

The interaction between post material and crown material remains unclear [2] as some authors reported better results when using high elastic modulus posts [21], while other authors reported better stress distribution when using low elastic modulus posts like glass fiber posts [5]. However, no differences in the stress distribution were found in some other studies [7].

So, our study aimed to evaluate the effect of different post and crown materials on stress distribution in endodontically treated maxillary central incisors. The null hypothesis was that there is no association between post and crown materials on the stress distribution and values in the tooth-restoration complex.

## Materials and methods

Model generation

Ten 3D virtual models of endodontically treated maxillary central incisors restored with a post and core were modeled using Geomagic DesignX 2016 (3D Systems, Inc.). The tooth was modeled with an overall length of 23.5 mm, crown length of 10.5 mm, and root length of 13 mm [22].

The tooth was then prepared to receive a full ceramic crown and a post and core restoration with no ferrule. Two-millimeter incisal edge reduction and a radial shoulder of 1 mm as a finish line were prepared. The prepared post space was 9 mm from the tooth cementoenamel junction (CEJ) in all models (approximately 2/3 of the root length). A four-millimeter cone with a 0.4 mm apical end was used to simulate the remaining gutta-percha.

A standardized double-tapered post resembles in shape size 3 D.T. Light-Post (RTD, France) (1.2 mm in diameter at the post apex and 2.18 mm in diameter at the post coronal end) was used in all models. The ten studied models were then divided into two equal groups according to the final crown material (zirconia and lithium disilicate). Each group consists of five models according to the post used: two prefabricated posts (zirconia and glass fiber) with composite cores, and three custom-made posts (gold, nickel-chrome, and zirconia).

**Figure 1:**
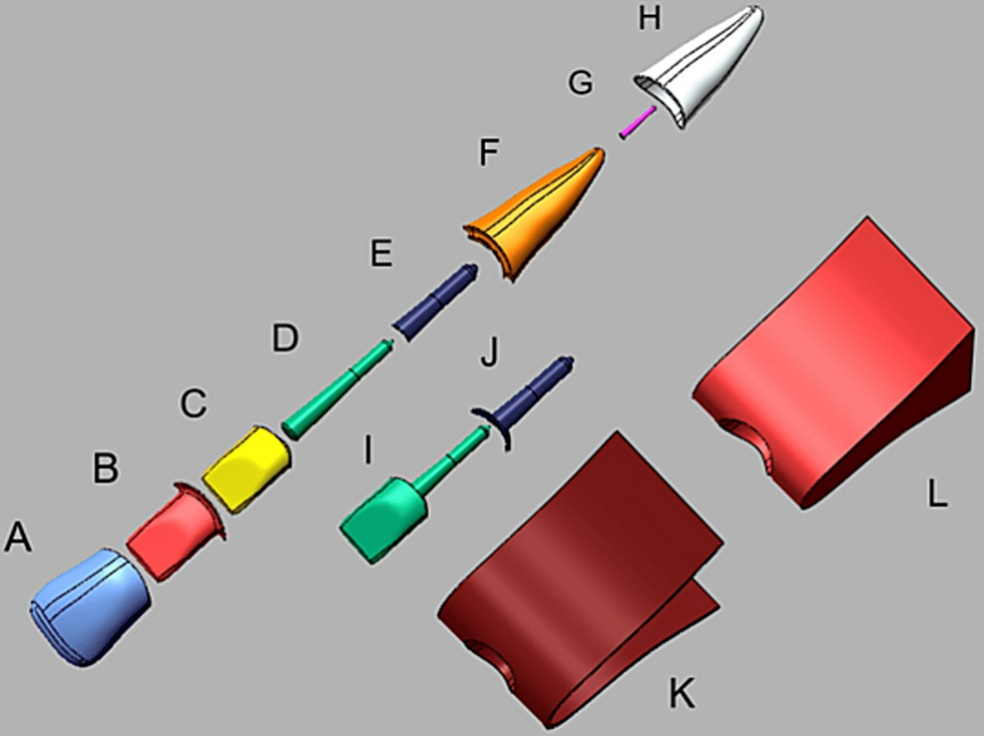
Model components: (A) crown, (B) crown-cement, (C) composite core, (D) prefabricated post, (E, J) post cement, (F) dentin, (G) gutta-percha, (H) periodontal ligament, (I) custom-made post, (K) cortical bone and (L) trabecular bone

A periodontal ligament of 0.2 mm was modeled on the root surface [23,24], as human periodontal ligament thickness ranged on average between 0.2 and 0.5 mm [25]. Supporting tissues including lamina dura of 0.25 mm [12], cortical bone of 0.5 mm thickness [7], and trabecular bone were also modeled. All posts and crowns were cemented with Panavia F2.0 (Kuraray, Japan) with 0.2 mm and 0.1 mm thickness, respectively (Figure 1).

Finite element analysis (FEA) setup

**Figure 2:**
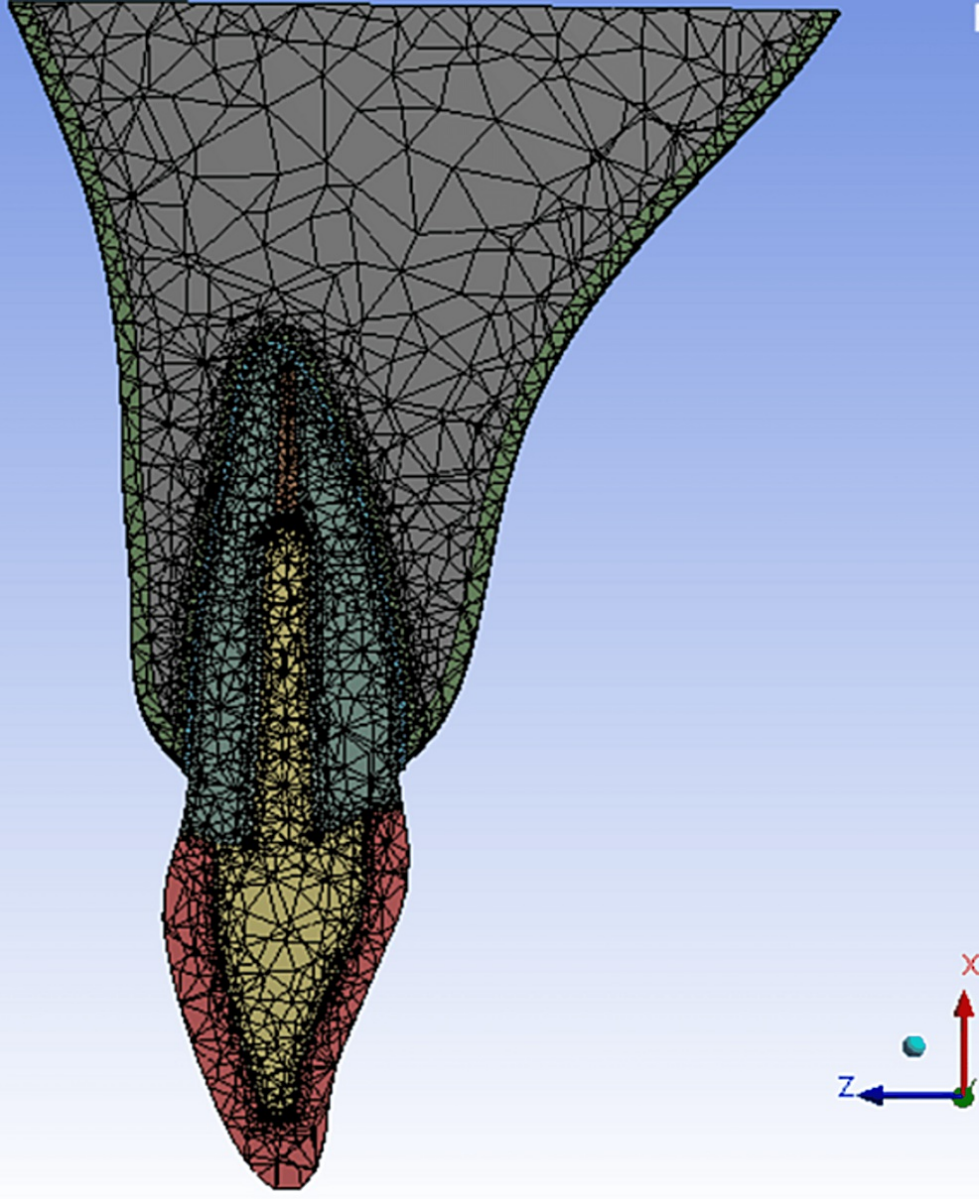
Meshing process

The geometries were exported to FEA solver software (Ansys 15, Ansys Inc.), and meshing was done using quadratic tetrahedral elements. Adaptive mesh refinements were done in regions of interest, and mesh convergence tests were conducted to obtain a mesh-independent solution which resulted in an average (442846) elements and (605533) nodes for the study models (Figure 2).

Young modulus and Poisson’s ratio were used to define material properties. Although many dental tissues like enamel and dentin show anisotropic properties. However, their coefficient of elasticity exhibits small differences in different directions due to the applied force [19]. Also, the periodontal ligament shows a nonlinear response to force. However, small differences in stress distribution were observed between linear and nonlinear periodontal ligament models [26]. So, this study assumed isotropic, linear elastic, and homogenous materials (Table 1) as an accepted simplification like other studies [7,25,27,28]. Interfaces between bodies were set to be fully bonded and were checked for possible gaps or voids.

**Table 1:** Elastic modulus and Poisson's ratio of materials used in the current study

Material	Elastic modulus in gigapascal (GPa)	Poisson’s ratio	Reference
Cortical bone	13.7	0.3	[12]
Trabecular bone	1.37	0.3	
Periodontal ligament	0.0689	0.45	
Dentin	18.6	0.31	
Gutta-percha	0.14	0.45	
Resin Composite	15.8	0.24	
Lithium disilicate	95	0.3	[7]
Zirconia	209.3	0.32	
Resin Cement (Panavia F2.0)	18.6	0.28	[21]
Glass fiber post	40	0.26	
Gold	100	0.31	[25]
Nickel-Chrome alloy	205	0.33	[8]

**Figure 3:**
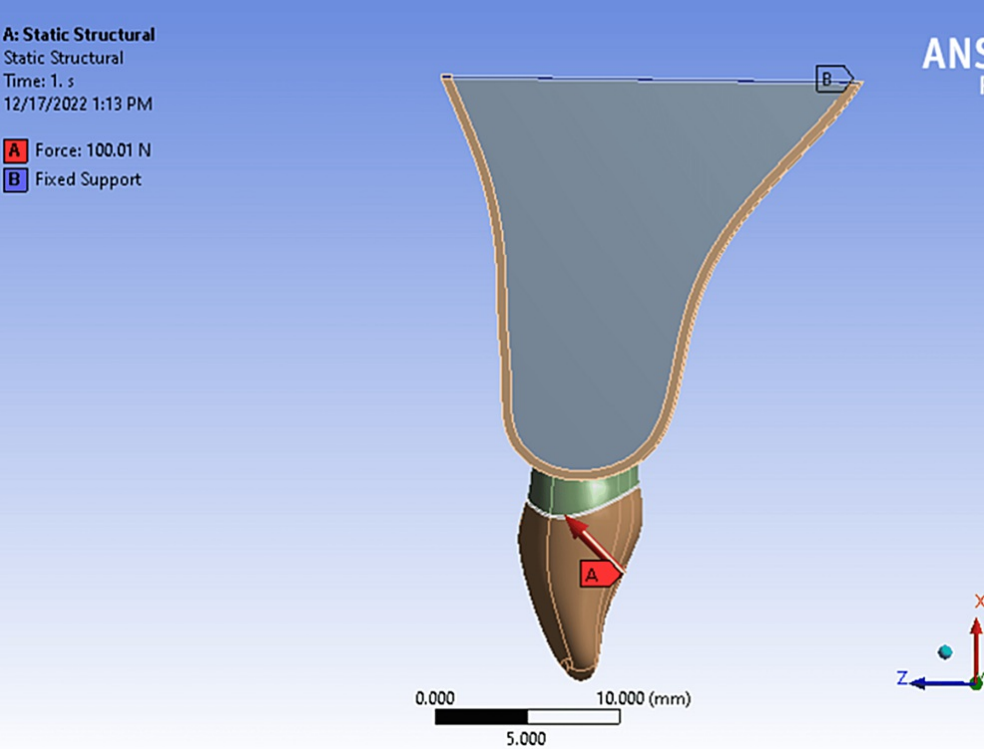
Force direction and boundary condition

An oblique static load (100 Newton) at 45° to the tooth longitudinal axis was applied to 5 mm^2^ contact area in the middle of the palatal surface of the crown. The bone base was fixated in all directions as a boundary condition (Figure 3). Each component of interest in the models was separated and sectioned in Bucco-palatal direction.

Equivalent von Mises stress was utilized for stress assessment as von Mises stress provides information about energy transmission within investigated bodies and helps in locating high-stress sites regardless of their nature (tensile or compressive) [4,12]. The results of each model component were obtained and color-coded images were used to better visualize the stress distribution where similar colors depict the same range of stresses generated and warmer colors (red) represent higher stresses.

## Results

**Figure 4:**
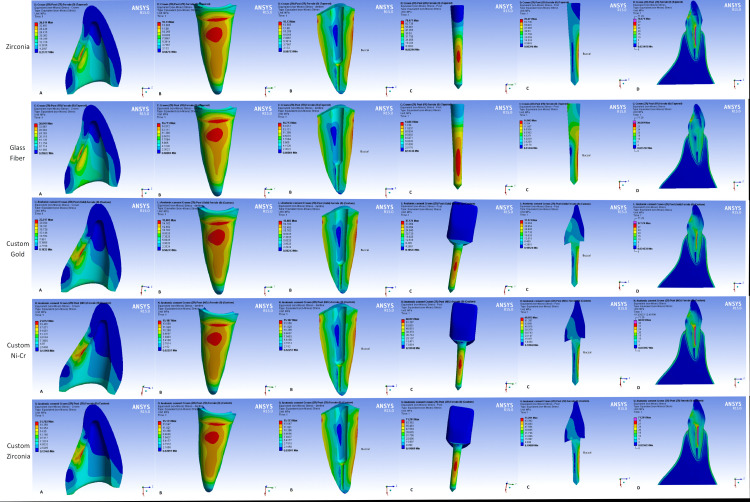
Stress distribution in (A) crown, (B) dentin, (C) post, (D) tooth-restoration complex in all models (crown material: zirconia)

**Figure 5:**
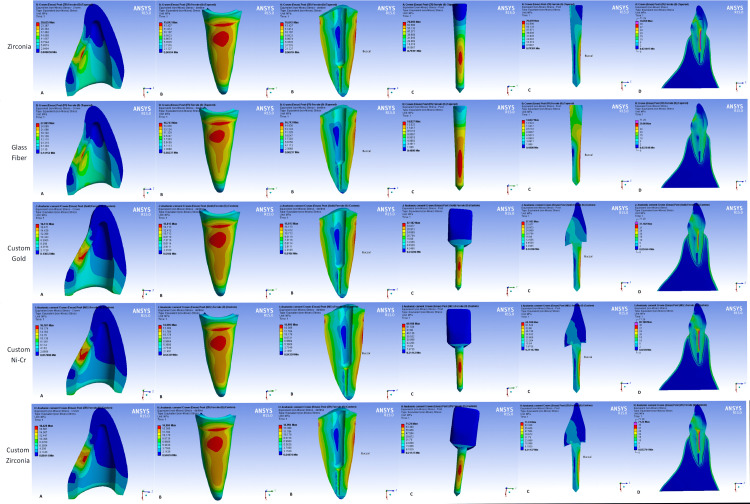
Stress distribution in (A) crown, (B) dentin, (C) post, (D) tooth-restoration complex in all models (crown material: lithium disilicate)

Maximum stress concentrations were observed in the middle third at the buccal sides of all the posts, regardless of their type. However, with glass fiber posts the stress extends to the apical third (Figures 4, 5).

Zirconia and metal cast posts (high elastic modulus posts) tend to concentrate more stress within their structures (Table 2). An additional stress concentration site was observed at the palatal junction of the post and core in custom-made posts and cores (Figures 4, 5).

**Table 2:** Maximum von Mises stress in megapascal (MPa) in the crown, dentin, and post in all modes

Post	Prefabricated				Custom-made					
Crown material	Lithuim disilicate		Zirconia		Lithuim disilicate			Zirconia		
Post material	Zirconia	Glass fiber	Zirconia	Glass fiber	Gold	Ni-Cr	Zirconia	Gold	Ni-Cr	Zirconia
Crown	28.62	27.08	36.51	36.05	18.51	18.19	18.63	23.52	21.91	21.76
Dentin	15.04	16.74	15.13	16.71	15.81	14.99	14.99	15.80	15.19	15.17
Post	70.66	14.03	70.47	14.04	37.18	69.19	71.24	37.17	68.92	71.29

Maximum stress concentration in root dentin was mainly observed in the cervical and middle buccal regions of the root regardless of the post type used. Glass fiber posts showed more homogeneous stress distribution in the tooth-restoration complex (Figures 4, 5). However, slightly more stress values were observed with glass fiber posts (low elastic modulus posts) in the cervical region (Table 2).

Stress concentrations in both crown materials were observed as expected at the force application site and were transferred to the intaglio palatal surface of the crowns (Figures 4, 5). Custom-made posts lead to a decrease in stress values in both crowns. However, more stress values were observed within zirconia crowns (Table 2).

## Discussion

Mechanical properties of dental restorations play a crucial role in the biomechanical performance of endodontically treated teeth restored with post-core systems. Therefore, the finite element method has become a useful tool to evaluate the stress distribution in the tooth-restoration complex, as it offers detailed numerical data within each component of the mathematical model. Moreover, it also helps in standardizing study parameters which are considered a hard task to accomplish in both in vitro and in vivo studies [27]. Although a failure in the oral cavity usually happens as a result of functional and parafunctional forces over a long period of time and would not happen because of high-stress concentration except in trauma situations [29]. The finite element method still provides valuable information regarding the mechanical behavior of tooth structures, dental materials, and their interaction. Moreover, fatigue failure would be expected to occur in high-stress locations [7].

Based on our study results the null hypothesis was rejected as post-material and crown material did influence the stress distribution and values in the tooth-restoration complex. In our study, stress distribution in root dentin was influenced by the post material as using glass fiber posts resulted in more homogenous stress distribution in the tooth-restoration complex. However slightly increased stress values in the cervical region in root dentin were observed in comparison to zirconia and metal cast posts which may be explained by the fact that glass fiber post flexibility permits more stress transfer to the cervical region of dentin, while more rigid zirconia and metal cast posts tend to concentrate more stress within their structures. This finding agreed with previous studies that examined the relation between post and core material and stress values in dentin, as they concluded that posts with high elastic modulus decreased stress in dentin despite higher stress concentration within the post and core system itself [8,12,21,28,30].

However, some other authors reported different results as they found that glass fiber posts caused less stress concentration in dentin compared to zirconia posts [31]. Also in a recent systematic review, the authors concluded that fiber posts induced less stress in root dentin compared to other posts [5]. However, these diverse outcomes may be the result of different material properties, boundary conditions, load application sites, magnitudes, and the type of FEA analysis utilized. It is noteworthy to mention that all models in this study showed stress accumulation in the cervical third of the root adjacent to the alveolar crest. This site is reported in many studies as a stress concentration area [12,21,31]. Different components with different elastic characteristics coming together in this area could explain this observation.

Gold posts and cores may be used with full ceramic crowns because of their yellowish color [17,25] but they are very expensive. Additionally, non-precious metal cast posts and cores cannot be used under high translucent full ceramic crowns (although they were virtually used in this study for comparative reasons) because of their dark color as they may cause darkening of the restoration gingival third and gingival margins [12]. With advancements in CAD/CAM technology and dental materials, zirconia custom-made posts and cores can be suggested as an alternative option, particularly in areas with high esthetic demand [25]. Our study results support this suggestion as both cast Ni-Cr post and core and custom-made zirconia post and core showed similar stress distribution and values.

Clinical studies focused primarily on prefabricated zirconia posts with direct (Composite resin) or indirect (ceramic heat-pressed) cores and reported mixing results [18,32]. Some concerns related to the use of prefabricated zirconia posts and cores in restoring endodontically treated teeth have been addressed such as bonding issues between posts and composite cores [12]. This problem can be overcome with the use of one-piece zirconia posts and cores. Moreover, the difficulty of retrieving posts whenever endodontic retreatment is needed or in cases of broken posts has also been discussed. Some authors suggested using non-bonding cements like zinc phosphate in luting custom-made zirconia posts and cores as retrieving posts luted with non-bonding cements using ultrasonic scalers are much easier than removing resin-bonded ones [25].

Recent studies evaluated the clinical outcomes of using CAD/CAM custom-made zirconia posts and cores and reported promising results [33,34]. Regarding crown material and its effect on stress distribution, both full ceramic crowns (zirconia, lithium disilicate) showed a similar stress distribution as the force applied on the crown's palatal surface caused stresses to accumulate on the intaglio palatal surface of the crown at the force application site. However, more stress values were observed within zirconia crowns, which may be explained by their higher stiffness which led to relatively higher stress concentrations compared to lithium disilicate crowns.

Both crown types in this study exhibited higher stress values when they were supported by composite resin cores and prefabricated posts which may be attributed to the lower elasticity coefficient of resin composite compared to that of integrated cores in custom-made posts used in this study. This finding agreed with the results of previous studies which concluded that ceramic crowns cemented to cast metal cores have lower failure rates because of lower stress concentration compared to crowns cemented to lower elastic modulus material like resin composite [35]. 

Based on our results crown material did not influence stress values in dentin, which contradicts the results drawn by Nokar and his colleagues [21] as they reported higher stress values in the cervical region of root dentin when using ceramic crowns compared to metal ceramic ones. However, using two different finish line designs (Chamfer with metal-ceramic crowns and radial shoulder with all ceramic crowns) and using different materials for crowns fabrication may explain these contradictory results.

As with all studies, this study has limitations such as the static nature of the force applied in this study which cannot reflect the real picture in the oral cavity as mastication forces have a more complex nature. Also, in this study, some simplifications were introduced as all materials were considered isotropic, linear elastic, and homogenous even though this could not be the situation in reality. These simplifications may lead to approximate results which may differ from the clinical situations. However, these simplifications are well-adopted in most finite element studies [7,25,27,28].

Moreover, many variables other than the ones addressed in this study like the presence of ferrule, post-design, post length, and post diameter may also affect the stress distribution and values in endodontically treated maxillary central incisors restored with post-core foundations. Finally, we should emphasize that the success of post-core restorations is not related to the restoration elastic modulus only as so many other factors could affect the success of these restorations like the presence of flaws within their structures, wear resistance, and oral cavity conditions. However, finite element studies can still predict the performance of different dental restorative materials and these studies should be considered complemental to clinical studies which provide definitive results.

## Conclusions

Within the limitations of this study, we can conclude that posts with high elastic modulus concentrate more stress within their structures. Although more homogenous stress distribution in the tooth-restoration complex was observed when using glass fiber posts. However, they slightly increase the stress values in root dentin. On the contrary, the crown material does not influence the stress distribution in root dentin. Both zirconia and lithium disilicate crowns showed similar stress concentration sites regardless of the post used. However, more stress values were observed within zirconia crowns. Additionally, crowns supported by high elastic modulus custom-made posts and cores exhibit lower stress values compared to crowns supported by composite resin cores and prefabricated posts. Custom-made zirconia posts and cores showed a similar stress distribution as non-precious cast metal posts and cores, so they may be used as a suitable option where esthetic is desired.
